# Disentangling the neural correlates of corticobasal syndrome and corticobasal degeneration with systematic and quantitative ALE meta-analyses

**DOI:** 10.1038/s41531-017-0012-6

**Published:** 2017-03-31

**Authors:** Franziska Albrecht, Sandrine Bisenius, Rodrigo Morales Schaack, Jane Neumann, Matthias L. Schroeter

**Affiliations:** 1 Max Planck Institute for Human Cognitive and Brain Sciences, 04103 Leipzig, Germany; 2 IFB Adiposity Diseases, Leipzig University Medical Center, 04103 Leipzig, Germany; 3 Clinic of Cognitive Neurology, Leipzig Research Center for Civilization Diseases, University of Leipzig & FTLD Consortium Germany, Ulm, Germany

## Abstract

Corticobasal degeneration is a scarce neurodegenerative disease, which can only be confirmed by histopathological examination. Reported to be associated with various clinical syndromes, its classical clinical phenotype is corticobasal syndrome. Due to the rareness of corticobasal syndrome/corticobasal degeneration and low numbers of patients included in single studies, meta-analyses are particularly suited to disentangle features of the clinical syndrome and histopathology. Using PubMed, we identified 11 magnetic resonance imaging studies measuring atrophy in 22 independent cohorts with 200 patients contrasted to 318 healthy controls. The anatomic likelihood estimation method was applied to reveal affected brain regions across studies. Corticobasal syndrome was related to gray matter loss in the basal ganglia/thalamus, frontal, parietal, and temporal lobes. In corticobasal degeneration patients, atrophy in the thalamus, frontal, temporal, and occipital lobes were found. Finally, in a conjunction analysis, the bilateral thalamus, the bilateral posterior frontomedian cortex, posterior midcingulate cortex and premotor area/supplementary motor area, and the left posterior superior and middle frontal gyrus/precentral gyrus were identified as areas associated with both, corticobasal syndrome and corticobasal degeneration. Remarkably, atrophy in the premotor area/supplementary motor area and posterior midcingulate/frontomedian cortex seems to be specific for corticobasal syndrome/corticobasal degeneration, whereas atrophy in the thalamus and the left posterior superior and middle frontal gyrus/precentral gyrus are also associated with other neurodegenerative diseases according to anatomic likelihood estimation method meta-analyses. Our study creates a new conceptual framework to understand, and distinguish between clinical features (corticobasal syndrome) and histopathological findings (corticobasal degeneration) by powerful data-driven meta-analytic approaches. Furthermore, it proposes regional-specific atrophy as an imaging biomarker for diagnosis of corticobasal syndrome/corticobasal degeneration ante-mortem.

## Differential diagnosis: imaging to tell the difference

Brain imaging could be used to distinguish between patients with corticobasal degeneration (CBD) and Parkinson's disease (PD). CBD is a rare condition caused by the gradual loss of brain cells in areas of the brain that link thinking to movement. The clinical features of CBD, referred to as corticobasal syndrome (CBS), are similar to those of patients with PD, but they progress differently. To aid earlier and more accurate diagnosis, Franziska Albrecht, at the Max Planck Institute for Human Cognitive and Brain Sciences, Germany, and colleagues reviewed 11 magnetic resonance imaging studies to find brain areas that are specifically affected in CBS/CBD patients. They show that cell loss in specific regions of the motor areas and frontomedian cortex is a hallmark of CBS/CBD, whereas cell loss in the thalamus and parts of the frontal/precentral gyrus were associated with other neurodegenerative diseases.

## Introduction

Recently, Armstrong *et al*.^1^ developed new diagnostic criteria underlining the use of the term corticobasal syndrome (CBS) only for clinical representation, whereas the term corticobasal degeneration (CBD) should label pathology-proven cases. CBS, as a scarce clinical phenotype, is associated with its classical histopathology, CBD, but also with other proteinopathies related to tau, amyloid, and transactive response DNA-binding protein.^2^ Diagnostic criteria of CBS associated with CBD include rigidity or akinesia, dystonia or myoclonus of limb, cortical sensory deficit, alien limb phenomena, or orrobuccal/limb apraxia.^1^ Interestingly, the disease most often affects the hand.^3^ When considering a patient having probable CBS with the pathology of CBD, four of the mentioned symptoms should be present asymmetrically. The patient should present at least two symptoms, which can be symmetric, for possible CBS. Regarding higher cortical features beside apraxia, the hallmark of CBS, additional cognitive impairments are often described in the literature.^1, 3^

Patients with CBD show widespread deposition of hyperphosphorylated 4-repeat tau in neurons and glia.^4^ Neuropathologic diagnostic criteria require Gallyas/tau-positive lesions, including neuronal inclusions, threads, coiled bodies, and astrocytic plaques as well as neuronal loss or additionally ballooned/achromatic neurons. Tau pathology can be found throughout gray and white matter, basal ganglia, diencephalon as well as rostral brainstem, while major lesions are found in superior frontal, pre-central/post-central and superior parietal gyri, the thalamus as well as the caudate nucleus.^5^ Encoded by the microtubule-associated protein tau (*MAPT*) gene, tau is responsible for microtubule assembly and stability. This function is controlled by the status of phosphorylation of tau protein. In the case of pathological dysfunction, binding affinity for microtubules decreases. Based on this tau-histopathology, several clinical phenotypes of CBD may evolve beside the most frequent syndrome CBS.^2^ This complex and highly variable clinical presentation underlines the challenge to accurately diagnose CBD in a living patient and develop specific diagnostic criteria.

Nowadays, physicians and scientists have to face and disentangle this wide range of clinical symptoms and distinct histopathologies leading to a broad clinicopathological heterogeneity. Aiming at a powerful, detailed description and disentanglement of clinical features and neurodegenerative patterns, here we performed a meta-analysis across voxel-based morphometry (VBM) imaging studies, applying structural magnetic resonance imaging (MRI) to detect disease-specific atrophy patterns. For the method, we applied anatomical likelihood estimate (ALE) meta-analyses that have been established as a standard tool for identifying prototypical neural correlates of neuropsychiatric diseases.^6–12^ The analysis was conducted separately for the clinical syndrome CBS, and for the (post-mortem) proven histopathology CBD to disentangle the interplay between clinical symptoms and underlying histopathology. Note that this approach enabled a strict dissociation and a subsequent comparison between the neural correlates of CBS and CBD. This distinction will be especially relevant for future treatment strategies aiming at specific proteinopathies.

We hypothesized that in CBS, severe atrophy will be found asymmetrically, mainly in the frontal lobe, due to the asymmetric occurrence of motor symptoms. According to findings in neuropathology, we hypothesized CBD lesions in the thalamus, caudate nucleus, superior frontal regions, regions around pre-central gyri/post-central gyri, and superior parietal regions. In addition to ALE meta-analyses conducted separately in CBS and CBD, we conducted a conjunction analysis to reveal overlapping regions, which is only specific for the disease combination of CBS/CBD.

## Results

**Fig. 1:**
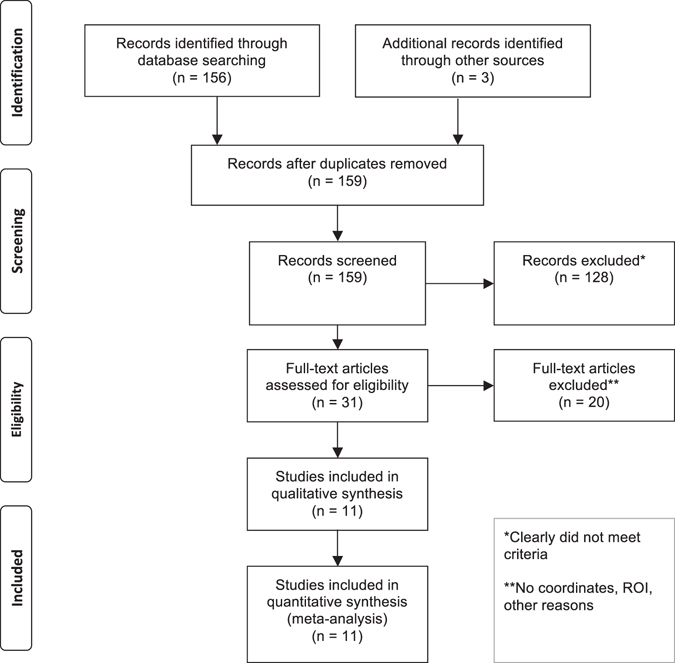
PRISMA statement flow diagram. Flow of information through different phases of the systematic literature search identifying the neural correlates of CBS and CBD according to the PRISMA statement, as suggested by Moher, Liberati, Tetzlaff and Altman.^13^ *ROI* region of interest

Figure 1 depicts the flow of information through the different phases of study selection process according to Moher *et al*.^13^ Details of the imaging studies for CBS and CBD that were included are summarized in Table 1 and Supplementary Table e-1. The meta-analysis integrated 11 MRI studies including 200 patients contrasted to 318 healthy controls. Note that two studies contained more than one cohort, leading to the inclusion of 22 independent patient cohorts. All studies involved reported either decreased gray matter volume or density, and none reported increases in gray matter volume or density in patients compared to control subjects. The CBS patient cohort included 184 patients with mean age of 66.3 ± 4.3 years (mean ± standard deviation), disease duration of 5.3 ± 2.0 years, and Mini-Mental State Examination (MMSE) score of 21.9 ± 2.8. The CBD cohort included 34 patients characterized by an age of 64.4 ± 5.9 years, disease duration of 7.0 ± 1.4 years, and a mean value in the MMSE of 23.9 ± 3.2. Unpaired Student’s *t*-tests indicate that there is no significant difference in age (*t* = −0.65, *p* = 0.54), and MMSE scores (*t* = 1.20, *p* = 0.27) between both cohorts. Note that mean disease duration was slightly, but non-significantly, longer in CBD than CBS in agreement with the post-mortem definition of CBD (*t* = 1.83, *p* = 0.09).

**Table 1:** Studies included in the meta-analyses that identified the neural correlates of CBS and CBD with MRI

Study	Pat. (N)	Clin. Syn.	Hist.	Cont. (N)	Gender (m/f)	Age (years)	Dis. Dur. (years)	MMSE	Diagnosis of Clin. Syn.	Notes
*Studies included in the CBS cohort*										
Borroni	20	CBS	NE	21	13/7	62.7 ± 8.0	2.0 ± 1.4	25.0 ± 3.6	Lang et al. 1994	a, b
Boxer	14	CBS	NE	80	4/10	64.6 ± 5.9	5.6 ± 1.7	19.2 ± 8.3	(own criteria)	a, b
Gross	6 out of 20	CBS	NE	12	9/11	67.4 ± 9.8	3.9 ± 2.0	22.7 ± 6.2	NR	b, *
Grossman	9	CBS	NE	12	NR	64.0 ± 7.0	3.4 ± 1.5	18.8 ± 7.4	(own criteria)	
Halpern	5 out of 13	CBS	NE	12	7/6	66.8 ± 10.2	4.2 ± 1.7	19.6 ± 5.1	Riley et al. 2000	
Huey	48 (1)	CBS	NE	14	25/23	66.0 ± 9.0	5.0	NR	Boeve et al. 2005	a, b
Koss	5 out of 10	CBS	NE	32	5/5	66.2 ± 8.3	NR	20.9 ± 6.1	Murray et al. 2007,(own criteria)	a, b, *
Pardini	25	CBS	NE	14	12/13	62.0 ± 9.0	4.0 ± 1.8	NR	NR	b
Lee	7 out of 9	CBS	AD	44	5/4	59.2	8.3	18.0	UCSF-MAC	b
	11 out of 14	CBS	CBD		4/10	66.0	6.7	23.9	UCSF-MAC	b
	4 out of 5	CBS	PSP		3/2	69.3	8.1	21.8	UCSF-MAC	b
	3 out of 5	CBS	FTLD-TDP		2/3	72.1	7.9	26.2	UCSF-MAC	b
	3 out of 5	CBS	Mixed (2)		4/1	75.8	5.0	25.0	UCSF-MAC	b
Whitwell	6	CBS	AD	24	2/4	60.0	7.0	26.0	Boeve et al. 2003	a, b
	7	CBS	CBD		1/6	68.0	6.0	20.0	Boeve et al. 2003	a, b
	6	CBS	PSP		3/3	69.0	7.0	27.0	Boeve et al. 2003	a, b
	5	CBS	FTLD-TDP		3/2	71.0	6.0	22.0	Boeve et al. 2003	a, b
Total CBS	184			265	102/110	66.3 ± 4.3	5.3 ± 2.0	21.9 ± 2.8		
*Studies included in the CBD cohort*										
Lee	3 out of 5	bvFTD	CBD	44	3/2	65.9	7.9	18.5	Neary et al. 1998	b
	11 out of 14	CBS	CBD		4/10	66.0	6.7	23.9	UCSF-MAC	b
	5 out of 7	EM	CBD		2/5	64.4	5.6	25.3	(own criteria)	b
	1	PCA	CBD		0/1	54.8	8.6	27.0	McMonagle et al. 2006	b
	4 out of 5	PNFA	CBD		1/4	71.0	5.6	25.0	Neary et al. 1998, Gorno-Tempini et al. 2004	b
Rankin	3 out of 5	bvFTD	CBD	53	3/2	61.4	7.6	22.0	Neary et al. 1998	a, b
Whitwell	7	CBS	CBD	24	1/6	68.0	6.0	20.0	Boeve et al. 2003	a, b
Total CBD	34			121	14/30	64.4 ± 5.9	7.0 ± 1.4	23.9 ± 3.2		
Total CBS + CBD(3)	200			318	111/124	65.8 ± 4.9	5.7 ± 2.0	22.5 ± 3.1		

Gender, age, disease duration, and MMSE scores are specified for patients (mean ± standard deviation). All MRI studies used 1.5T (except for * = 3T). Clinical data are reported for all patients, whereas in four studies, MRI analyses were performed in a subcohort. Total mean scores were calculated without Rankin and Whitwell because they reported data as median. Data on references of included studies and diagnostic criteria are available in the Supplementary data (Supplementary Table e-1). Underline signifies the total score.

^a^ Correction for multiple comparisons

^b^ modulated

(1) Only 5 CBD cases were proven by pathology. (2) Mixed cases showed features of PSP, CBD and FTLD-TDP mixed with possible AD. (3) CBS–CBD cohorts of Lee and Whitwell were only used once to calculate total CBS + CBD

*AD* Alzheimer’s disease, *bvFTD* behavioral variant frontotemporal dementia, *CBD* corticobasal degeneration, *CBS* corticobasal syndrome, *Clin. Syn.* clinical syndrome, *Con.* controls, *Dis. Dur.* disease duration, *EM* executive-motor, *f* female, *FTLD-TDP* frontotemporal lobar degeneration with TAR DNA-binding protein 43 inclusions, *Hist.* histopathology, *m* male, *MMSE* Mini-Mental State Examination, *N* number of subjects, *NE* not examined, *NR* not reported, *Pat.* patients, *PCA* posterior cortical atrophy, *PNFA* progressive nonfluent aphasia, *PSP* progressive supranuclear palsy, *UCSF-MAC* University of California, San Francisco Memory and Aging Center criteria for CBS

**Fig. 2:**
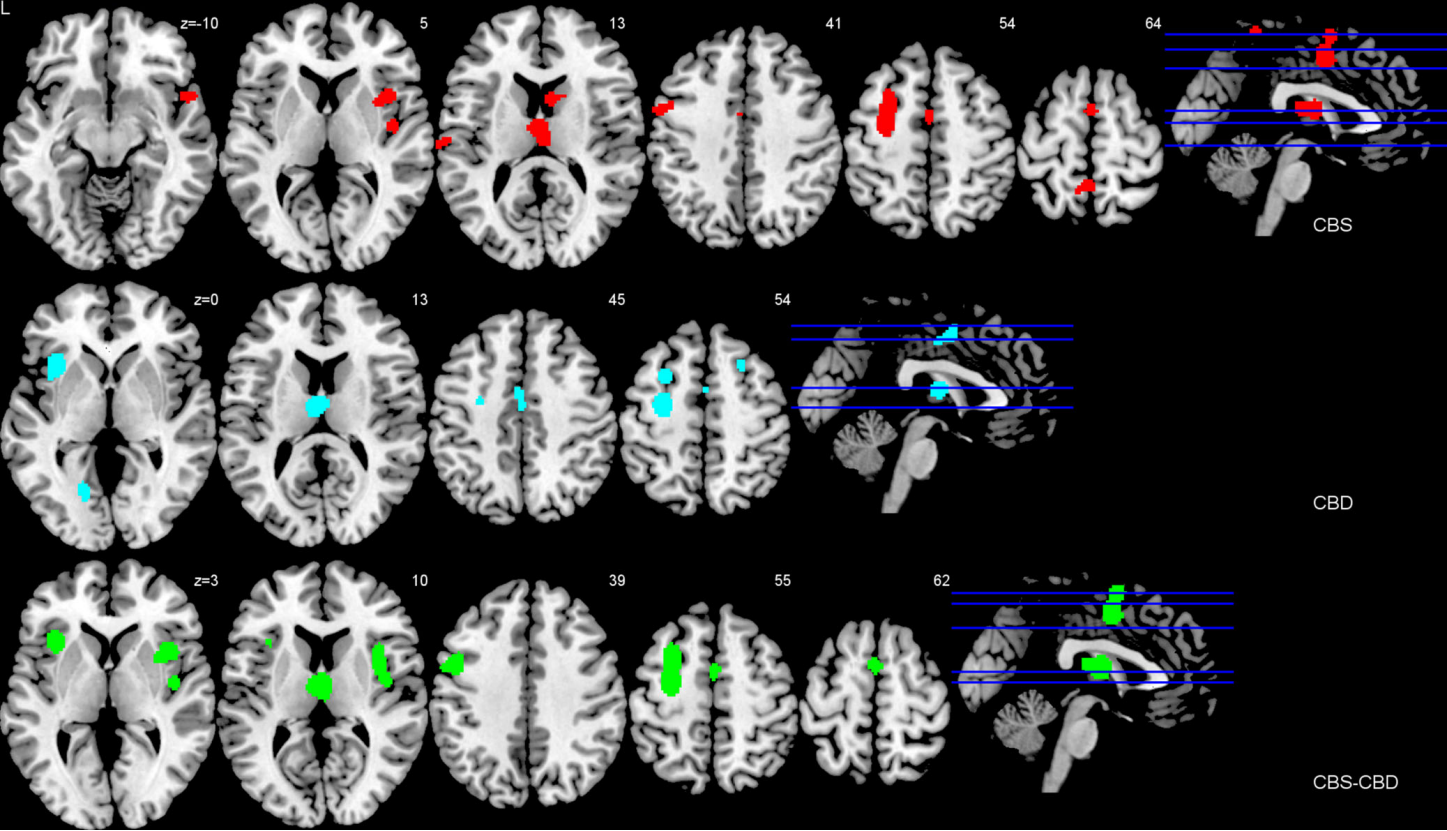
Impaired regions in CBS and CBD. Impaired brain regions in CBS and CBD in comparison with healthy control subjects–anatomical likelihood estimates meta-analyses. Atrophy was measured by MRI. The CBS analysis (*red*) included 184 patients from 17 cohorts contrasted to 265 healthy subjects. The CBD analysis (*blue*) included 34 patients from seven cohorts contrasted to 121 healthy subjects. The analysis of the combined cohort of CBS and CBD (*white*) included 200 patients contrasted to 318 healthy control subjects. Coordinates are reported in MNI space. *L* left

The upper part of Fig. 2 and Supplementary Table e-2 illustrate the results of the ALE meta-analysis identifying the neural correlates of CBS. The analysis across MRI studies revealed regional atrophy in the frontal, parietal and temporal lobe, and the basal ganglia/thalamus. More specifically, CBS affects the thalamus, the superior precuneus/postcentral gyrus and the posterior frontomedian cortex, posterior midcingulate cortex and premotor area/supplementary motor area bilaterally. In the right hemisphere, CBS is associated with atrophy in the caudate nucleus, the anterior superior temporal gyrus, the anterior superior insula, the claustrum, as well as putamen, and the posterior insula. In the left hemisphere, the meta-analysis additionally identified the precentral gyrus, posterior superior frontal sulcus and middle/superior frontal gyrus, a cluster in the inferior frontal junction area/posterior inferior frontal sulcus and precentral gyrus, and one cluster in the inferior postcentral sulcus/superior temporal gyrus.

The results of the ALE meta-analysis that investigated the neural correlates of CBD are displayed in the middle part of Fig. 2 and Supplementary Table e-2. Note that patients with histopathological CBD included several clinical symptoms beside CBS (see Table 1). CBS was clinically observed in only 53% (18 out of 34 cases). In general, the analysis across MRI studies revealed atrophy in the thalamus, frontal, temporal and occipital lobes. In detail, post-mortem proved CBD affects the thalamus, and the posterior frontomedian cortex, posterior midcingulate cortex and premotor areas/supplementary motor areas bilaterally. The right hemisphere shows atrophy in the posterior superior frontal sulcus and middle/superior frontal gyrus, while the left hemisphere shows gray matter loss in the medial/lateral occipitotemporal gyrus, the precentral gyrus, posterior superior frontal sulcus and middle/superior frontal gyrus, the posterior frontal sulcus and middle frontal sulcus, and the anterior insula.

The lower part of Fig. 2 and Supplementary Table e-2 show the results of the ALE meta-analysis combining all CBS and CBD patients. Generally, the results of this analysis revealed that the basal ganglia/thalamus and frontal lobe are affected. In detail, atrophy was again identified in the thalamus, and the posterior frontomedian cortex, posterior midcingulate cortex and premotor areas/supplementary motor areas bilaterally. Further, atrophy was detected in the right insula and claustrum/putamen. Finally, the left hemisphere showed gray matter loss in the inferior frontal junction/posterior inferior frontal gyrus and precentral gyrus, the precentral gyrus/posterior superior frontal sulcus and middle/superior frontal gyrus, and the anterior superior insula.

**Fig. 3:**
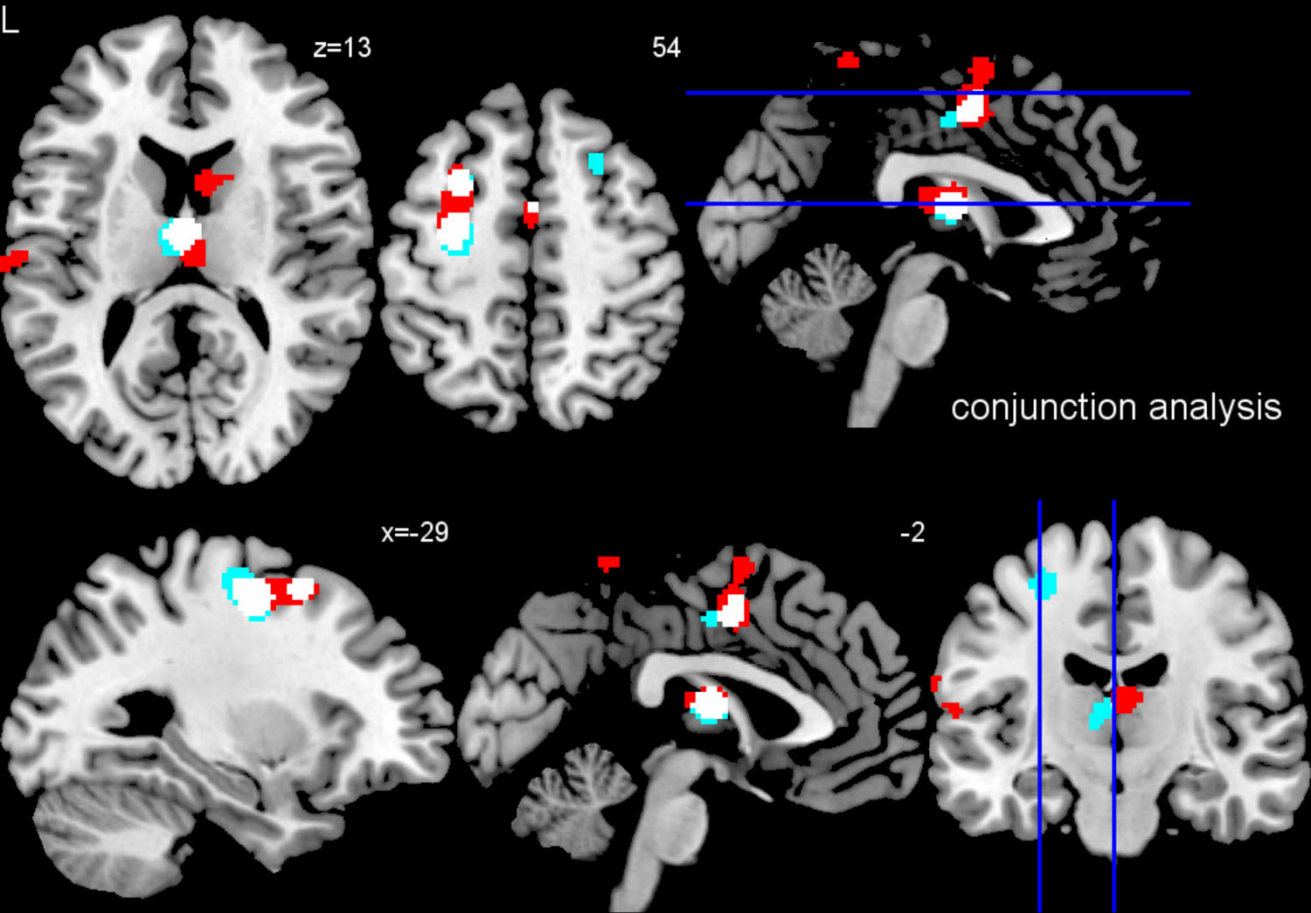
Conjunction analysis. Conjunction analysis for impaired brain regions in CBS and CBD in comparison with healthy control subjects–anatomical likelihood estimates meta-analyses. White clusters indicate overlap of both meta-analyses (CBS and CBD). Atrophy was measured by MRI. Coordinates are reported in MNI space. *L* left

Finally, a conjunction analysis was performed by overlaying the results of the two separate meta-analyses on CBS and CBD to identify brain regions involved in both, CBS and CBD. Results are displayed in Fig. 3, where white shading indicates overlap of both analyses. The analysis revealed that four clusters (1) in the bilateral thalamus, (2) in the posterior frontomedian cortex/posterior midcingulate cortex and premotor area/supplementary motor area, (3) in the left posterior superior frontal sulcus and middle frontal gyrus/precentral gyrus, and (4) in the left posterior superior frontal sulcus and middle frontal gyrus were consistently atrophic in both cohorts, CBS and CBD.

## Discussion

To our knowledge, here we present the first powerful data-driven meta-analyses identifying and comparing the neural correlates of CBS and CBD for MRI measuring atrophy in comparison with healthy control subjects. The design enabled high validity and statistical power by including 200 patients with this rare disease and 318 healthy control subjects. Additionally, the inclusion criterion of whole brain imaging studies for all meta-analyses guaranteed a data-driven approach.

Our results reveal that CBS is characterized by gray matter loss in the basal ganglia/thalamus, frontal, parietal and temporal lobes. In CBD patients, atrophy in the thalamus, frontal, temporal and occipital lobes was found. The latter results on CBD agree with Dickson *et al*.,^4^ who described histopathologically atrophy and tau-immunoreactive lesions in the superior frontal/parietal, pre- and postcentral gyri, the caudate nucleus, the putamen, and the thalamus in CBD.

Most remarkably, in a conjunction analysis focusing on common brain regions affected by both, CBS and CBD, our study identified four brain areas with significant atrophy: (1) the bilateral anterior thalamus, and (2) the bilateral posterior frontomedian cortex, posterior midcingulate cortex and premotor area/supplementary motor area, and, in the left hemisphere, (3) the posterior superior frontal sulcus and middle frontal gyrus/precentral gyrus, and (4) the left posterior superior frontal sulcus and middle frontal gyrus. As these regions obviously constitute the core hubs for CBS/CBD according to our meta-analyses, we want to discuss them now in more detail. With regard to disease specificity, we will compare results of our ALE meta-analysis with structural MRI ALE meta-analyses of other neurodegenerative diseases (Table 2). Note that the differential diagnosis between CBS/CBD, progressive supranuclear palsy, multiple system atrophy, Lewy body disease, and Parkinson’s disease is of paramount interest, as all of these might be associated with an (atypical or typical) Parkinson’s syndrome.

**Table 2:** Meta-analytical evidence for biomarker specificity of regional atrophy in CBS/CBD as measured with MRI

Disease	Study	Anatomical region				
		Thalamus	Bl. fronto-median and midcingulate cortex	Bl. premotor area and supplementary motor area	L. pre-central gyrus	L. superior frontal sulcus and middle frontal gyrus
Parkinson’s syndromes						
Parkinson’s disease	^15, 16^	−	−	−	+	+
Atypical Parkinson’s syndromes						
CBD and syndrome	Present meta-analysis	+	+ (posterior)	+	+	+
Progressive supranuclear palsy	^15^	+	−	−	+	−
Multiple system atrophy	^16^	+	−	−	−	−
Lewy body dementia	^40*^	(−)	(−)	(−)	(−)	(−)
Alzheimer’s disease	^9, 12, 41^	+	−	−	−	−
Behavioral variant frontotemporal dementia	^8–11^	−	+ (anterior)	−	−	−
Primary progressive aphasias						
Nonfluent/ agrammatic variant (progressive non-fluent aphasia)	^9, 10, 42^	−	−	−	−	+
Semantic variant (semantic dementia)	^9, 10, 42, 43^	−	−	−	−	−
Logopenic variant (logopenic aphasia)	^42^	−	−	−	−	−

Meta-analyses were conducted by calculating ALEs in the gray matter. Other methods, such as effect-size signed differential mapping, were not taken into account to avoid a bias of different methods.

*Only one study by Zhong et al.^40^ applying anisotropic effect size version of signed differential mapping (AES-SDM) was included, because no ALE meta-analysis has been available to date.

*Bl* bilateral, *L* left, *NR* not reported to date

### Thalamic and left precentral atrophy is not specific for CBS/CBD

Although the thalamus was altered in both, CBS and CBD, changes in thalamic structure do not seem to be an exclusive imaging biomarker for CBS/CBD. Other neurodegenerative diseases, such as Alzheimer’s disease, progressive supranuclear palsy, behavioral variant frontotemporal dementia, or Huntington’s disease are also characterized by thalamic atrophy.^14^ Atrophy in the thalamus was also confirmed in recent ALE meta-analyses in Alzheimer’s disease, progressive supranuclear palsy, and multiple system atrophy.^9, 12, 15, 16^ Furthermore, thalamic volume also shrinks in healthy people throughout life independent from and prior to total volume loss.^17^

In our conjunction analysis, the region of the left posterior superior frontal sulcus and middle frontal gyrus/precentral gyrus also showed significant atrophy. Remarkably, atrophy was located anterior to the right hand area in the precentral gyrus,^18^ fitting well with the clinical finding that the hand is most often impaired in CBS.^3^ The precentral gyrus encompasses parts of primary motor and premotor cortex. Dorsal premotor regions are responsible for promoting movements and response selection depending on spatial cues.^19^ Ventral premotor regions regulate hand movement to manipulate and grasp objects, and contain functions related to cognition such as the comprehension of actions. Goldenberg^20^ suggested that atrophy in ventral regions of the left hemisphere is correlated with apraxia, in particular imitating hand postures and using or pantomiming the use of mechanical tools. Clinically, CBS is most frequently linked to apraxia^21^–an impairment in higher cortical features concerning skillful motoric movement without motoric deficits per se.

With regard to other neurodegenerative diseases (Table 2), recent meta-analyses also show atrophy in the left middle frontal gyrus in Parkinson’s disease, and in the nonfluent/agrammatical variant of primary progressive aphasia, while atrophy in the left precentral gyrus is shown in Parkinson’s disease and progressive supranuclear palsy.^9, 10, 15, 16^ This leads to the conclusion that atrophy in these brain regions is not disease-specific for CBS/CBD.

### Atrophy in premotor area/supplementary-motor area and posterior midcingulate/frontomedian cortex is specific for CBS/CBD

According to the conjunction analysis, the premotor areas and supplementary motor areas showed atrophy in both CBS and CBD. These regions are essential for linking cognition to action^22^ and their atrophy can lead to the alien limb phenomenon, which is part of the diagnostic criteria for CBS. Although Parkinson’s disease patients display less activation or a loss of neurons in this region,^22^ ALE meta-analyses have never identified these brain regions as affected by atrophy in other neurodegenerative diseases, including Parkinson’s disease (Table 2; in particular^15, 16^).

Additionally, the conjunction analysis revealed significant gray matter loss in the posterior frontomedian and midcingulate cortex in CBS/CBD. Although former ALE meta-analyses have shown atrophy in behavioral variant frontototemporal dementia in the anterior frontomedian and midcingulate cortex,^8–11^ the respective location was much more posterior in CBS/CBD without any overlap. In sum, atrophy in the premotor area/supplementary motor area and posterior midcingulate/frontomedian cortex seems to be specific for CBS/CBD, suggesting these brain regions as candidates for diagnostic and differential diagnostic imaging biomarkers in CBS/CBD in contrast to other neurodegenerative diseases (Table 2).

### Study’s limitations

Our study identified consistent findings of gray matter volume changes in CBS and CBD, but it also has its limitations. A main deficit of all included studies is the lack of a common standard in using special diagnostic criteria for evaluating CBS, where some studies used idiosyncratic criteria based on clinical expertise (Table 1). Therefore, slightly differently included clinical symptoms and, at least partly, imprecise diagnoses are possible. However, due to the meta-analytical approach, this confounding effect should be minor and not bias our results decisively. Armstrong *et al*.^1^ built a foundation for new consensus criteria, which should be employed in clinical studies to help overcome the problem of not strictly comparable diagnosis. In future, it will be possible to include only studies based on these new criteria in meta-analyses. Due to low incidence and ethical issues, MRI studies on autopsy proven cases of CBD are rare. Hence, results of our CBD analysis should be handled with caution due to the small cohort of patients. CBS/CBD patients are characterized by asymmetrical atrophy and symptoms. Unfortunately, the original studies did not separate their analyses into patients suffering from left or right-sided clinical symptoms, although Armstrong *et al*. (2013) underlined the importance of symptom laterality in the new diagnostic criteria. Studies just reported local maxima of atrophy from whole CBS/CBD cohorts. Thus, conducting separate meta-analyses for left and right sided CBS was not possible. An important contributing factor could be the preference for using either the right or left hand. We were not able to control for handedness due to unreported data. One should also consider that there are methodological differences between VBM studies, for instance, field-strength of scanners, processing protocols, or data modulation. However, these differences would not have confounded our results, because the ALE method only takes maxima into account and not cluster sizes. During the literature search, we did not find enough ^18^F-fluorodeoxyglucose positron emission tomography (FDG-PET) or diffusion tensor imaging (DTI) studies, which reported coordinates in stereotactic space, to conduct meaningful meta-analyses. Thus, our meta-analyses had to be limited to structural MRI atrophy data. We recommend extending meta-analyses to other imaging modalities in the future, when more studies are available. It is well-known that for different imaging modalities regional alterations might be dissociated in time and space.^8, 12, 23, 24^

Furthermore, specificity and sensitivity of the suggested disease-specific brain regions in differentiation of neurodegenerative diseases like CBS/CBD have to be validated in large, preferably multi-centric, and independent patient cohorts. These patient cohorts should ideally provide information about clinical and imaging data as well as surrogate markers for histopathology from serum and cerebrospinal fluid or even post mortem validation. This approach has already been successfully applied to other neurodegenerative diseases such as Alzheimer’s disease and frontotemporal lobar degeneration.^25, 26^ Validating results of ALE meta-analyses in independent cohorts is also necessary due to the fact that these meta-analyses generally include maxima and not cluster sizes of the various imaging studies. Consequently, single studies might have shown that dementia diseases may be regionally more unspecific than the present meta-analyses suggest.

## Conclusion

The meta-analyses build a foundation for a better disentanglement of the clinical phenotype CBS, and the histopathological type CBD. Results suggest atrophy in the premotor area/supplementary motor area and posterior midcingulate/frontomedian cortex as an imaging biomarker for diagnosis of CBS or CBD ante-mortem.

## Materials and methods

### General study selection

Assuring validity and quality, the meta-analysis was conducted according to preferred reporting items for systematic reviews and meta-analyses (PRISMA) guidelines^13^ (also see www.prisma-statement.org). To identify adequate studies, PubMed was used applying the following search strategy: (corticobasal degeneration OR CBD OR corticobasal syndrome OR CBS) AND (voxel* OR gray matter OR VBM). Studies were included if they fulfilled the following criteria: (1) peer-reviewed, (2) diagnosis according to established diagnostic criteria, (3) original study, (4) comparison with age-matched healthy control group, (5) results normalized to a stereotactic space such as the Talairach^27^ or the Montreal Neurological Institute’s^28^ (MNI) reference system and respective coordinates available, (6) whole brain study. Region-of-interest analyses or case studies were excluded to prevent any a priori regional assumptions. Since we did not find enough imaging studies that applied FDG-PET or DTI, which reported coordinates in stereotactic space, studies were limited to structural MRI atrophy data. We concentrated on reporting gray matter atrophy studies because only two studies analyzed white matter changes. Literature search was performed between November 2014 and January 2015 reviewing all studies regardless of their publication date. During this process, we contacted two study authors and obtained data on maxima from one study.

### Statistical analyses

To extract the neural correlates of CBS and CBD, we applied the ALE meta-analysis method.^29–32^ This method was first invented for meta-analyses of functional imaging studies with psychological stimulation of participants (here called activation likelihood estimate). Later, this approach was extended to imaging studies during rest investigating atrophy or hypometabolism.^6–12^

In 2009, Eickhoff *et al*.^33^ presented a revised analysis algorithm. The underlying method transforms extracted peaks into Gaussian probability distributions around these coordinates. The estimation of the width of these Gaussian probability distributions is adapted for each study according to the number of included subjects based on empirical estimates of between-subject and between-template variability. The resulting ALE maps are later combined across studies and tested against the null hypothesis of a random spatial distribution between the modeled maps. In this step, regions where empirical ALE values are higher than those expected by chance are identified. The resulting map is thresholded with a customized threshold to report only cluster that exceed the number of voxels corresponding to 5% possible false positives.

The analyses were performed in BrainMap Ginger ALE 2.3^34^ using extracted peaks of atrophy in CBS/CBD studies. Coordinates reported in the stereotactic space of Talairach and Tournoux were transformed into the stereotactic MNI space using the Lancaster transform implemented in Ginger ALE 2.3.^35^ As an error corrector for multiple testing we applied the conservative false discovery rate nonparametric *p*-value threshold (FDR pN) correction^30, 36^ with a threshold of *p* < 0.05. Clusters with a minimum size of 374 mm^3^ are reported. Recently, Eickhoff, Laird, Fox, Lancaster, and Fox (2016)^37^ reported about implementation errors in determining thresholds to account for multiple comparisons in older GingerALE software distributions. Note that ALE scores and peak locations are unaffected by these errors. We compare our results to other meta-analyses using also these versions of GingerALE. This way, we ensure homogeneity and comparability to other previous meta-analyses on neurodegenerative diseases. Future studies shall apply the new corrected GingerALE software versions homogeneously for different neurodegenerative diseases.

To disentangle the neural correlates for CBS/CBD specifically, we performed data analyses in three cohorts: (1) cohort of clinically evaluated CBS patients (CBS cohort), (2) cohort of histopathologically proven CBD patients (CBD cohort), and (3) cohort including all CBS and CBD data (CBS + CBD cohort). Additionally, we conducted a conjunction analysis between the results of the separate meta-analyses on CBS and CBD in order to reveal regions that were consistently atrophied in both cohorts.

For purposes of visualization, results were imported into MRIcron.^38^

To reveal possible differences in age, disease duration, and MMSE scores between groups, we performed Student’s *t*-tests in R^39^ with a significance threshold at *p* < 0.05.

### Potential bias

Several methods were applied to reduce the risk of bias of single studies and across studies. To overcome the problem of a bias toward specific brain areas, only studies using a quantitative automated whole brain analysis were included. Studies reporting results of region-of-interest approaches were not considered. To exclude any impact of age as a confounding factor on our results, only studies comparing patients with age-matched healthy controls were included into the meta-analysis. Studies investigating brain atrophy compared to other neurodegenerative diseases were also excluded to avoid any bias due to divergent control groups. According to PRISMA statement, two investigators performed literature search and selection separately (FA and RMS). As proposed in Eickhoff’s *et al*.^33^ new algorithm, the number of patients included in each study was taken into account in data synthesis. This leads to a balanced analysis. Although studies with negative findings might theoretically have been omitted, this publication bias is unlikely, because CBS and CBD patients generally show severe atrophy in comparison with healthy control subjects.

## Electronic supplementary material

Supplemental Data
