# Supplementary material for: Disentangling the neural correlates of corticobasal syndrome and corticobasal degeneration with systematic and quantitative ALE meta-analyses
Source: NPJ Parkinsons Dis. 2017 Mar 31;3:12. doi: 10.1038/s41531-017-0012-6 (PMC5459811; doi:10.1038/s41531-017-0012-6)
Supplement: Supplementary file 1 — Supplemental Data [file 41531_2017_12_MOESM1_ESM.docx]

**SUPPLEMENTAL DATA**

Table e-1 References of diagnostic criteria. Listed are all studies included in the meta-analysis and the diagnostic criteria they referred to.

| **Study** | **Clinical Syndrome** | | **Histo-pathology** | **Diagnosis of Clinical Syndrome** |
| --- | --- | --- | --- | --- |
| *Studies without post-mortem analysis* | | | | |
| Borroni et al. 2008 ^e1^ | CBS | NE | | Lang et al. 1994 ^e2^ |
| Boxer et al. 2006 ^e3^ | CBS | NE | | Neurologist (own criteria(2)) |
| Gross et al. 2010 ^e4^ | CBS | NE | | Neurologist (criteria not mentioned) |
| Grossman et al. 2004 ^e5^ | CBS | NE | | Neurologist (using published criteria) |
| Halpern et al. 2004 ^e6^ | CBS | NE | | Riley et al. in Litvan 2000 ^e7^ |
| Huey et al. 2009 ^e8^ | CBS | NE | | Boeve et al. in Litvan 2005 ^e9^ |
| Koss et al. 2010 ^e10^ | CBS | NE | | Murray et al. 2007 (own criteria (3)) ^e11^ |
| Pardini et al. 2009 ^e12^ | CBS | NE | | Neurologist (criteria not mentioned) |
| *Studies with post-mortem analysis* | | | | |
| Lee et al. 2010 ^e15^ | CBS | AD | | UCSF-MAC Criteria for CBS(4) |
|  | CBS | CBD | | UCSF-MAC Criteria for CBS |
|  | CBS | PSP | | UCSF-MAC Criteria for CBS |
|  | CBS | FTLD-TDP | | UCSF-MAC Criteria for CBS |
|  | CBS | Mixed (1) | | UCSF-MAC Criteria for CBS |
| Whitwell et al. 2010 ^e13^ | CBS | AD | | Boeve et al. 2003 ^e14^ |
|  | CBS | CBD | | Boeve et al. 2003 |
|  | CBS | PSP | | Boeve et al. 2003 |
|  | CBS | FTLD-TDP | | Boeve et al. 2003 |
| Lee et al. 2010 | bvFTD | CBD | | Neary et al. 1998 ^e16^ |
|  | CBS | CBD | | UCSF-MAC Criteria for CBS |
|  | EM | CBD | | Neurologist (own criteria(5)) |
|  | PCA | CBD | | McMonagle et al. 2006 ^e17^ |
|  | PNFA | CBD | | Neary et al. 1998, Gorno-Tempini et al. 2004 ^e18^ |
| Rankin et al. 2011 ^e19^ | bvFTD | CBD | | Neary et al. 1998 |
| Whitwell et al. 2010 | CBS | CBD | | Boeve et al. 2003 |
| (1) Mixed cases showed features of PSP, CBD and FTLD-TDP mixed with possible AD.  (2) Slow progressive course, asymmetric limb or axial rigidity, aphasia/visuospatial impairment/neglect/apraxia, dystonia/myoclonus/cortical sensory loss/alien limb. (3) Insidious onset and gradual progression of apraxia, cortical sensory loss, extrapyramidal disorder. (4) UCSF-MAC University of California, San Francisco Memory and Aging Center Criteria further explained in Lee et al. 2010 ^e15^ (5) Predominance of motor features including dystonia, axial or appendicular rigidity, progressive loss of limb function and problems in executive functions. | | | | |

**Table e-2** Results of the anatomical likelihood estimates meta-analyses identifying the neural correlates of corticobasal syndrome and corticobasal degeneration using magnetic resonance imaging.

| **Region** | **BA** | **Lat.** | **x** | **y** | **z** | **ALE** | **Vol.** |
| --- | --- | --- | --- | --- | --- | --- | --- |
| **CBS** | | | | | | | |
| Precentral gyrus/posterior superior frontal sulcus & middle/superior frontal gyrus | 4/6/8 | L. | -30 | -6 | 52 | .0206 | 3040 |
|  |  |  | -26 | 6 | 54 | .0168 |  |
| Thalamus |  | Bl. | 2 | -18 | 14 | .0182 | 1920 |
|  |  |  | 0 | -8 | 14 | .0175 |  |
| Posterior frontomedian cortex, posterior midcingulate cortex, pre- & supplementary motor area | 4/6/24/31 | L. | 0 | 2 | 64 | .0138 | 1736 |
|  |  |  | -2 | 0 | 46 | .0133 |  |
| Inferior frontal junction area/posterior inferior frontal sulcus, precentral gyrus | 6/9/44 | L. | -48 | 8 | 34 | .0142 | 1216 |
|  |  |  | -58 | 2 | 42 | .0118 |  |
| Anterior superior insula, claustrum, putamen | 13/14 | R. | 42 | 12 | 0 | .0112 | 1032 |
|  |  |  | 34 | 8 | 4 | .0105 |  |
| Caudate nucleus |  | R. | 8 | 10 | 14 | .0156 | 568 |
|  |  |  | 16 | 12 | 12 | .0081 |  |
| Posterior insula | 13 | R. | 44 | -8 | 6 | .0134 | 480 |
| Superior precuneus/postcentral gyrus | 7 | Bl. | -2 | -46 | 66 | .0123 | 480 |
|  |  |  | -10 | -52 | 62 | .0091 |  |
| Anterior superior temporal gyrus | 22/38 | R. | 54 | 12 | -12 | .0129 | 456 |
| Inferior postcentral sulcus/superior temporal gyrus | 2/40-43 | L. | -68 | -20 | 14 | .0113 | 456 |
|  |  |  | -68 | -18 | 20 | .0109 |  |
|  |  |  | -62 | -18 | 10 | .0094 |  |
| **CBD** | | | | | | | |
| Precentral gyrus/posterior superior frontal sulcus & middle/superior frontal gyrus | 4/6 | L. | -28 | -8 | 52 | .0148 | 2352 |
| Thalamus |  | Bl. | -4 | -10 | 12 | .0146 | 1720 |
| Anterior insula | 14/15 | L. | -36 | 20 | 2 | .0112 | 1568 |
| Posterior frontomedian cortex, posterior midcingulate cortex, pre- & supplementary motor area | 4/6/24/31 | L. | 0 | 0 | 50 | .0073 | 776 |
|  |  |  | 0 | -10 | 44 | .0065 |  |
| Medial/lateral occipitotemporal gyrus | 19 | L. | -18 | -64 | -2 | .0085 | 552 |
|  |  |  | -16 | -68 | -2 | .0085 |  |
| Posterior superior frontal sulcus & middle frontal gyrus | 6/8 | L. | -26 | 10 | 54 | .0088 | 512 |
| Posterior superior frontal sulcus & middle/superior frontal gyrus | 6/8 | R. | 24 | 16 | 56 | .0080 | 392 |
| **CBS + CBD** | | | | | | | |
| Precentral gyrus/posterior superior frontal sulcus & middle/superior frontal gyrus | 4/6/8 | L. | -28 | -6 | 52 | .0246 | 4280 |
|  |  |  | -26 | 6 | 54 | .0186 |  |
| Insula & claustrum/putamen | 13-15 | R. | 44 | -8 | 6 | .0163 | 3664 |
|  |  |  | 38 | 14 | 6 | .0137 |  |
|  |  |  | 38 | 4 | 10 | .0118 |  |
| Thalamus |  | Bl. | 0 | -8 | 14 | .0242 | 3072 |
|  |  |  | 2 | -16 | 14 | .0197 |  |
| Inferior frontal junction area/posterior inferior frontal sulcus, precentral gyrus | 4/6/9/44 | L. | -48 | 8 | 34 | .0155 | 2072 |
|  |  |  | -58 | 2 | 42 | .0119 |  |
| Posterior frontomedian cortex, posterior midcingulate cortex,  pre- & supplementary motor area | 4/6/24/31 | Bl. | 0 | 2 | 62 | .0152 | 2024 |
|  |  |  | -2 | 0 | 46 | .0134 |  |
| Anterior superior insula | 15 | L. | -36 | 20 | 4 | .0171 | 1376 |
| Clusters below an anatomical likelihood estimate (ALE) threshold p < 0.05, false discovery rate (FDR), are listed. Coordinates are in MNI space. Abbreviations: BA Brodmann area, Bl. bilateral, CBD corticobasal degeneration, CBS corticobasal syndrome, Cl. Cluster, L. left, Lat. lateralization, MNI Montreal Neurological Institute, R. right, Vol. volume in mm^3^. | | | | | | | |

**REFERENCES**

e1. Borroni B, Garibotto V, Agosti C, et al. White matter changes in corticobasal degeneration syndrome and correlation with limb apraxia. Arch Neurol 2008;65:796-801.

e2. Lang AE, Riley DE, Bergeron C. Cortical-basal ganglionic degeneration. Neurodegenerative diseases Philadelphia: WB Saunders 1994:877-894.

e3. Boxer AL, Geschwind MD, Belfor N, et al. Patterns of brain atrophy that differentiate corticobasal degeneration syndrome from progressive supranuclear palsy. Arch Neurol 2006;63:81-86.

e4. Gross RG, Ash S, McMillan CT, et al. Impaired information integration contributes to communication difficulty in corticobasal syndrome. Cogn Behav Neurol 2010;23:1-7.

e5. Grossman M, McMillan C, Moore P, et al. What's in a name: voxel-based morphometric analyses of MRI and naming difficulty in Alzheimer's disease, frontotemporal dementia and corticobasal degeneration. Brain 2004;127:628-649.

e6. Halpern CH, Glosser G, Clark R, et al. Dissociation of numbers and objects in corticobasal degeneration and semantic dementia. Neurology 2004;62:1163-1169.

e7. Riley DE, Lang AE. Clinical diagnostic criteria. In: Litvan I, Goetz CG, Lang AE, eds Corticobasal degeneration and related disorders Phila- delphia: Lippincott Williams & Wilkins 2000:29-34.

e8. Huey ED, Pardini M, Cavanagh A, et al. Association of ideomotor apraxia with frontal gray matter volume loss in corticobasal syndrome. Arch Neurol 2009;66:1274-1280.

e9. Boeve BF. Corticobasal degeneration: the syndrome and the disease. In: Litvan, I, editor Atypical Parkinsonian Disorders: Clinical and Research Aspects Totowa, NJ: Humana Press Inc 2005:309-334.

e10. Koss S, Clark R, Vesely L, et al. Numerosity impairment in corticobasal syndrome. Neuropsychology 2010;24:476-492.

e11. Murray R, Neumann M, Forman MS, et al. Cognitive and motor assessment in autopsy-proven corticobasal degeneration. Neurology 2007;68:1274-1283.

e12. Pardini M, Huey ED, Cavanagh AL, Grafman J. Olfactory function in corticobasal syndrome and frontotemporal dementia. Arch Neurol 2009;66:92-96.

e13. Whitwell JL, Jack CR, Jr., Boeve BF, et al. Imaging correlates of pathology in corticobasal syndrome. Neurology 2010;75:1879-1887.

e14. Boeve BF, Lang AE, Litvan I. Corticobasal degeneration and its relationship to progressive supranuclear palsy and frontotemporal dementia. Ann Neurol 2003;54 Suppl 5:S15-19.

e15. Lee SE, Rabinovici GD, Mayo MC, et al. Clinicopathological correlations in corticobasal degeneration. Ann Neurol 2011;70:327-340.

e16. Neary D, Snowden JS, Gustafson L, et al. Frontotemporal lobar degeneration: a consensus on clinical diagnostic criteria. Neurology 1998;51:1546-1554.

e17. McMonagle P, Deering F, Berliner Y, Kertesz A. The cognitive profile of posterior cortical atrophy. Neurology 2006;66:331-338.

e18. Gorno-Tempini ML, Dronkers NF, Rankin KP, et al. Cognition and anatomy in three variants of primary progressive aphasia. Ann Neurol 2004;55:335-346.

e19. Rankin KP, Mayo MC, Seeley WW, et al. Behavioral variant frontotemporal dementia with corticobasal degeneration pathology: phenotypic comparison to bvFTD with Pick's disease. J Mol Neurosci 2011;45:594-608.
